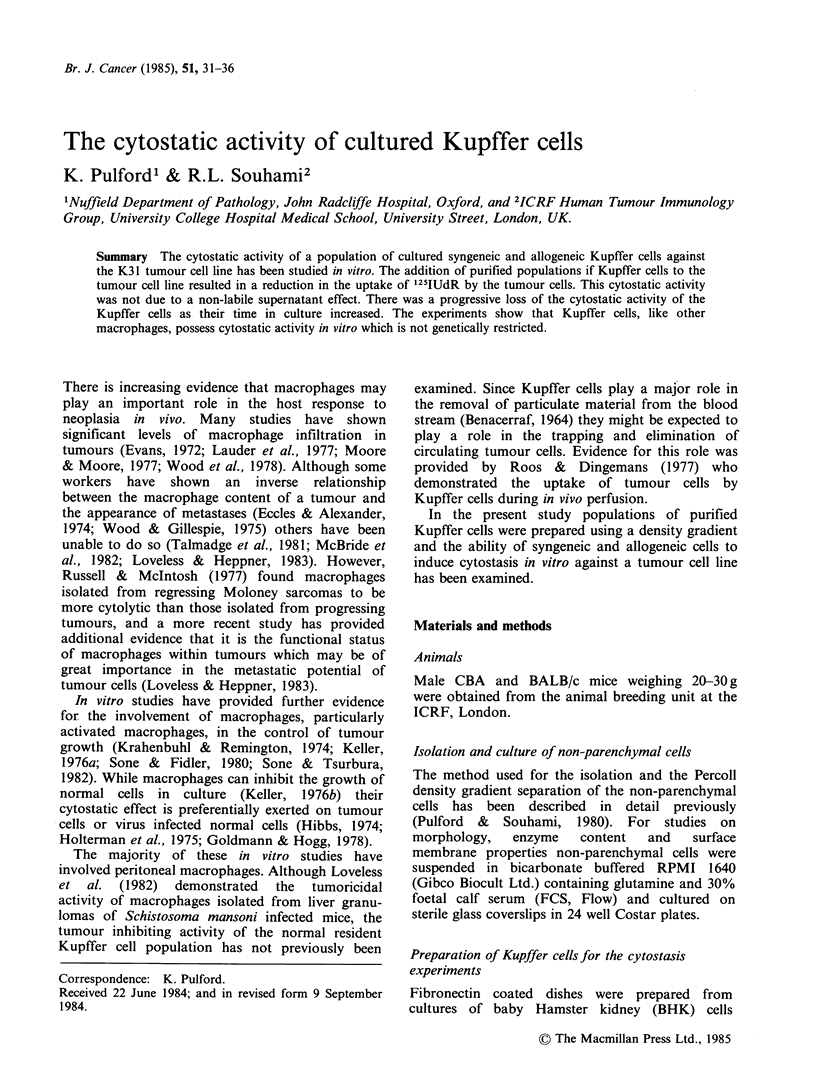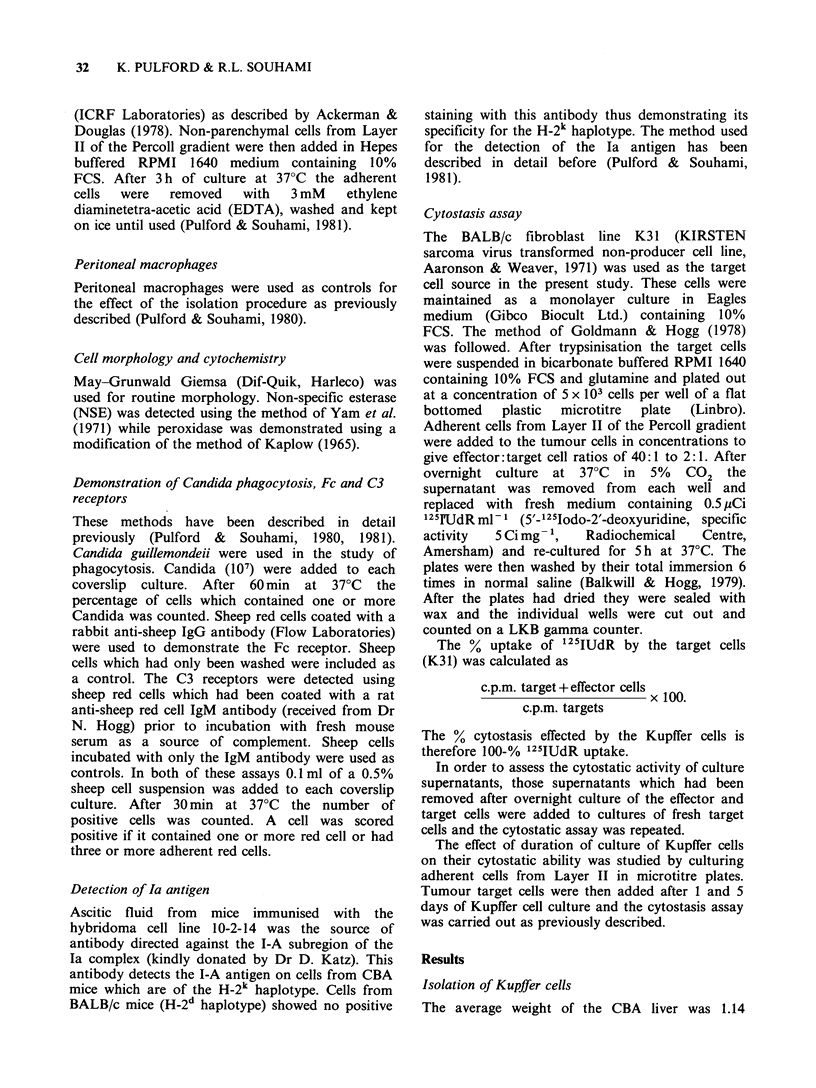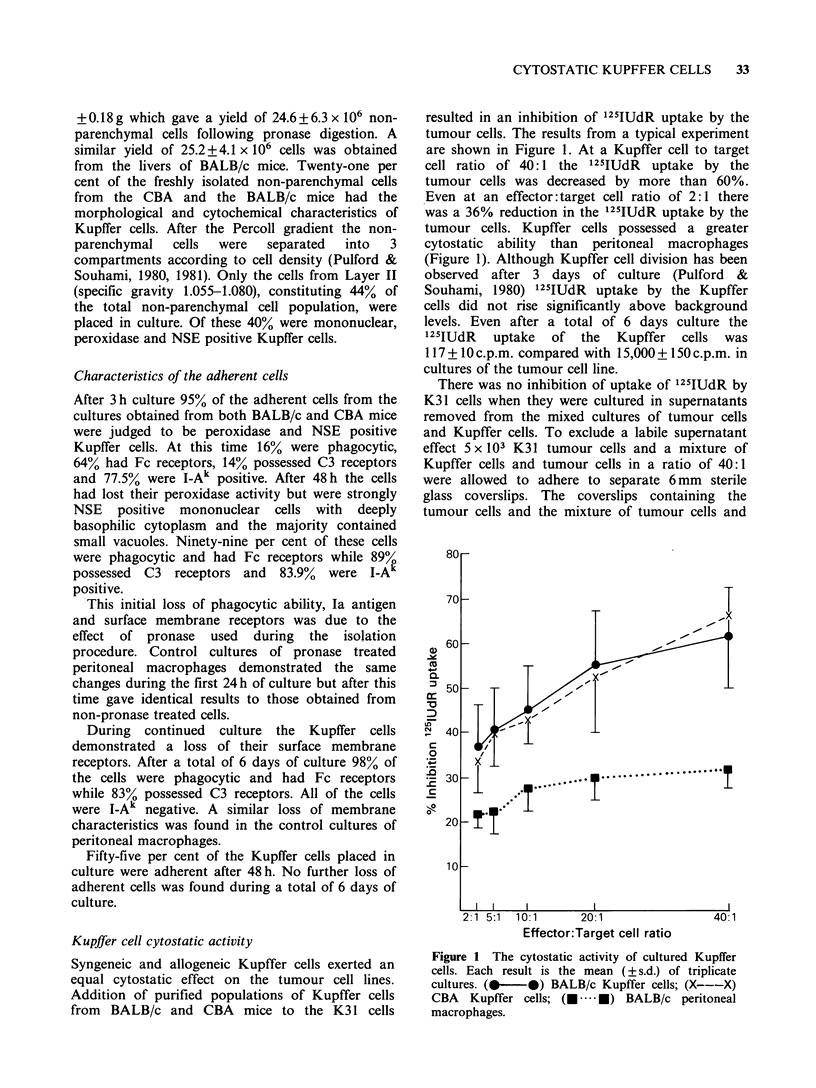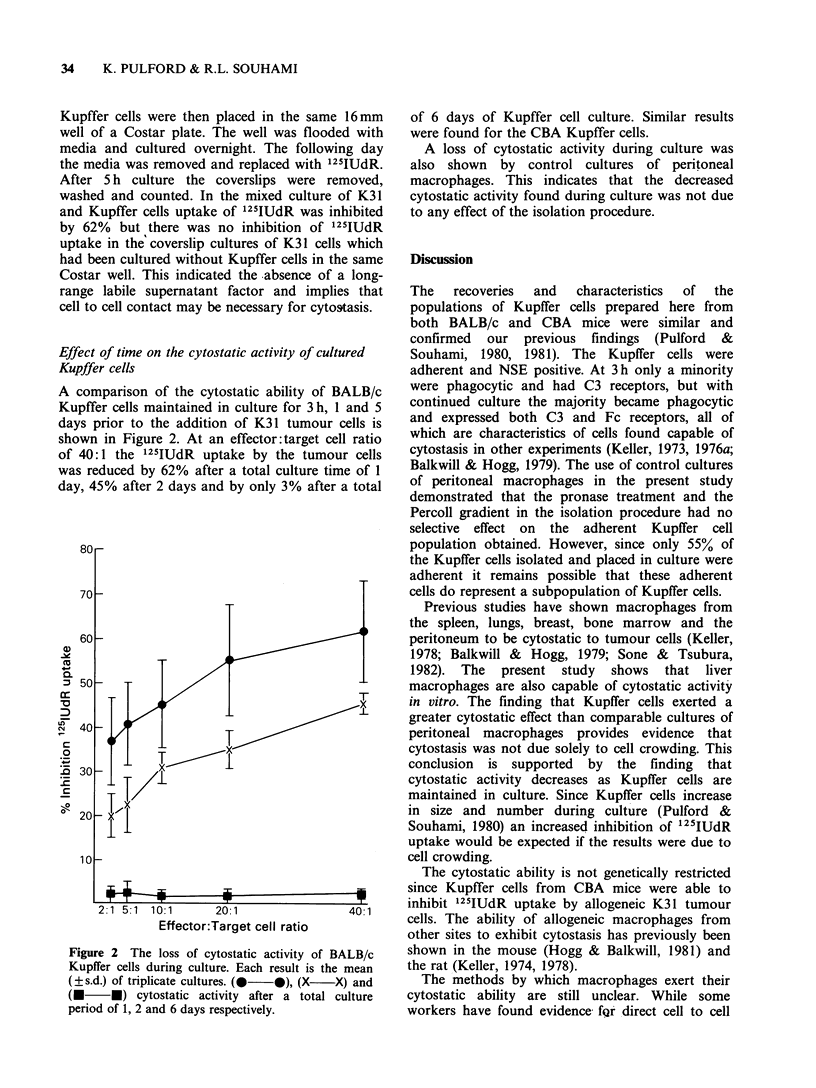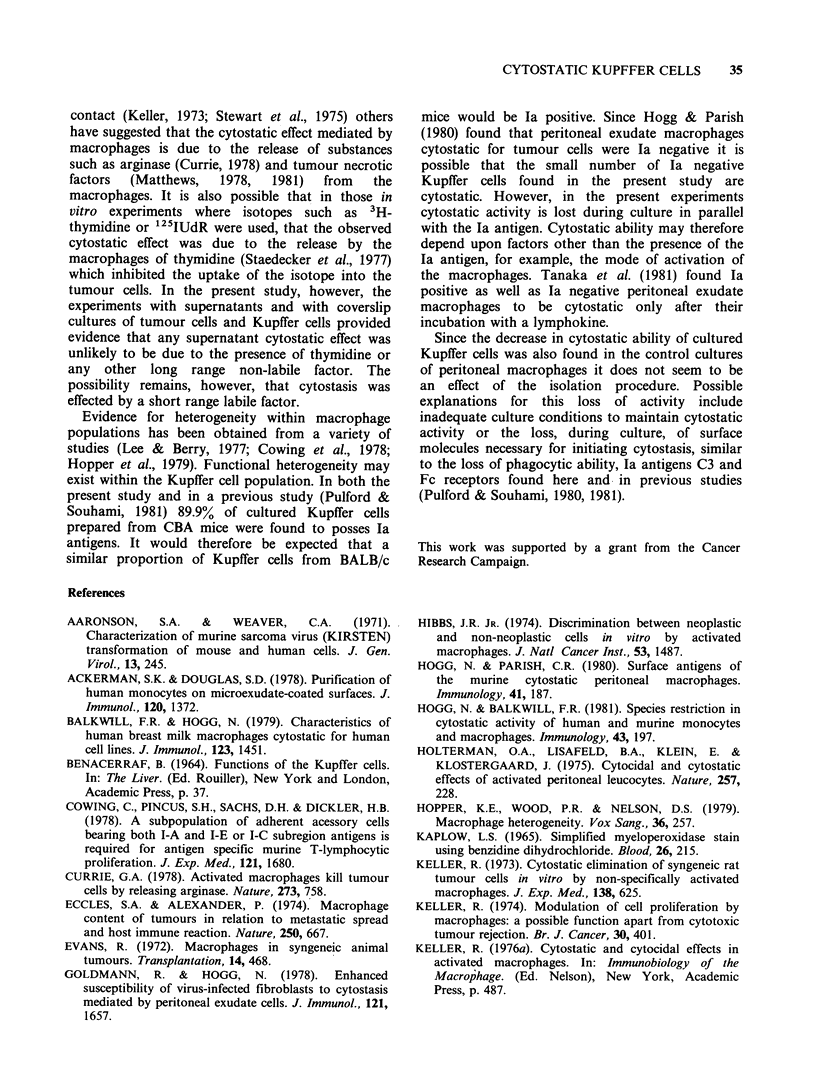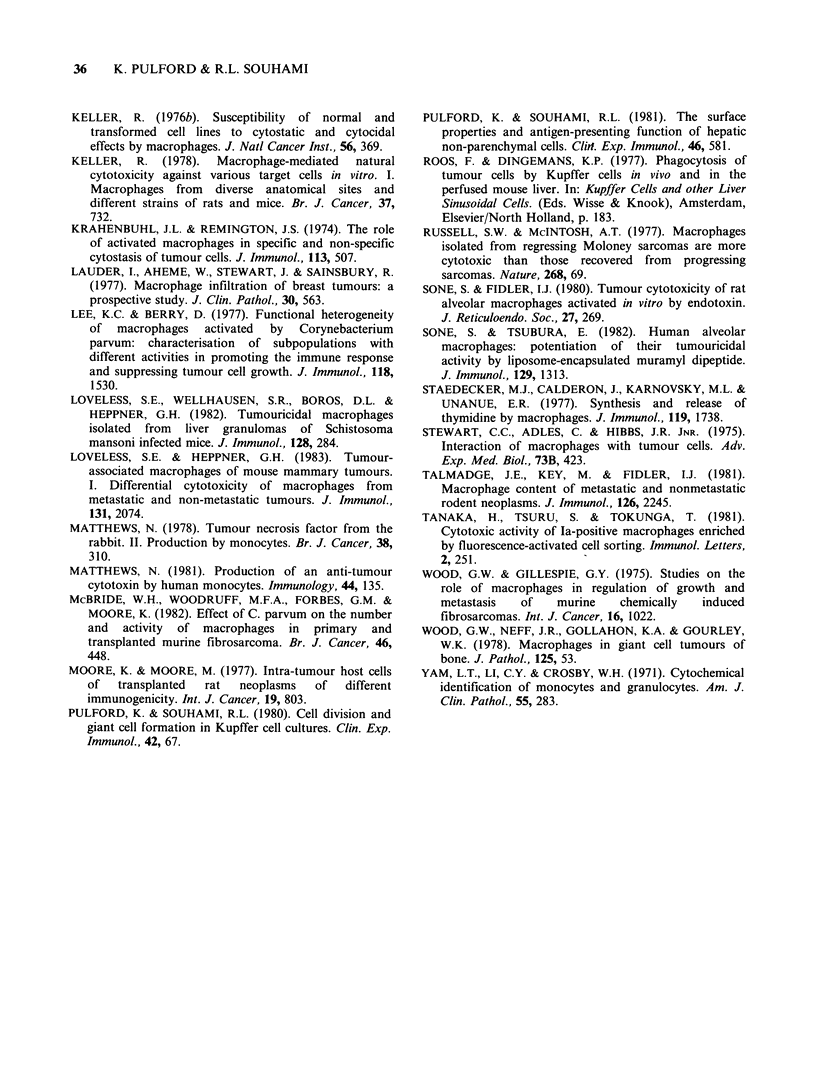# The cytostatic activity of cultured Kupffer cells.

**DOI:** 10.1038/bjc.1985.5

**Published:** 1985-01

**Authors:** K. Pulford, R. L. Souhami

## Abstract

The cytostatic activity of a population of cultured syngeneic and allogeneic Kupffer cells against the K31 tumour cell line has been studied in vitro. The addition of purified populations if Kupffer cells to the tumour cell line resulted in a reduction in the uptake of 125IUdR by the tumour cells. This cytostatic activity was not due to a non-labile supernatant effect. There was a progressive loss of the cytostatic activity of the Kupffer cells as their time in culture increased. The experiments show that Kupffer cells, like other macrophages, possess cytostatic activity in vitro which is not genetically restricted.


Br. J. Cancer (1985), **51**, 31–36

### The cytostatic activity of cultured Kupffer cells

K. Pulford[1] & R.L. Souhami[2]

[1]*Nuffield Department of Pathology, John Radcliffe Hospital, Oxford, and* [2]*ICRF Human Tumour Immunology Group, University College Hospital Medical School, University Street, London, UK.*

**Summary** The cytostatic activity of a population of cultured syngeneic and allogeneic Kupffer cells against the K31 tumour cell line has been studied *in vitro*. The addition of purified populations if Kupffer cells to the tumour cell line resulted in a reduction in the uptake of $^{125}$IUdR by the tumour cells. This cytostatic activity was not due to a non-labile supernatant effect. There was a progressive loss of the cytostatic activity of the Kupffer cells as their time in culture increased. The experiments show that Kupffer cells, like other macrophages, possess cytostatic activity *in vitro* which is not genetically restricted.

There is increasing evidence that macrophages may play an important role in the host response to neoplasia *in vivo*. Many studies have shown significant levels of macrophage infiltration in tumours (Evans, 1972; Lauder *et al.*, 1977; Moore & Moore, 1977; Wood *et al.*, 1978). Although some workers have shown an inverse relationship between the macrophage content of a tumour and the appearance of metastases (Eccles & Alexander, 1974; Wood & Gillespie, 1975) others have been unable to do so (Talmadge *et al.*, 1981; McBride *et al.*, 1982; Loveless & Heppner, 1983). However, Russell & McIntosh (1977) found macrophages isolated from regressing Moloney sarcomas to be more cytolytic than those isolated from progressing tumours, and a more recent study has provided additional evidence that it is the functional status of macrophages within tumours which may be of great importance in the metastatic potential of tumour cells (Loveless & Heppner, 1983).

*In vitro* studies have provided further evidence for the involvement of macrophages, particularly activated macrophages, in the control of tumour growth (Krahenbuhl & Remington, 1974; Keller, 1976*a*; Sone & Fidler, 1980; Sone & Tsurbura, 1982). While macrophages can inhibit the growth of normal cells in culture (Keller, 1976*b*) their cytostatic effect is preferentially exerted on tumour cells or virus infected normal cells (Hibbs, 1974; Holterman *et al.*, 1975; Goldmann & Hogg, 1978).

The majority of these *in vitro* studies have involved peritoneal macrophages. Although Loveless *et al.* (1982) demonstrated the tumoricidal activity of macrophages isolated from liver granulomas of *Schistosoma mansoni* infected mice, the tumour inhibiting activity of the normal resident Kupffer cell population has not previously been examined. Since Kupffer cells play a major role in the removal of particulate material from the blood stream (Benacerraf, 1964) they might be expected to play a role in the trapping and elimination of circulating tumour cells. Evidence for this role was provided by Roos & Dingemans (1977) who demonstrated the uptake of tumour cells by Kupffer cells during *in vivo* perfusion.

In the present study populations of purified Kupffer cells were prepared using a density gradient and the ability of syngeneic and allogeneic cells to induce cytostasis *in vitro* against a tumour cell line has been examined.

### Materials and methods

### *Animals*

Male CBA and BALB/c mice weighing 20–30 g were obtained from the animal breeding unit at the ICRF, London.

### *Isolation and culture of non-parenchymal cells*

The method used for the isolation and the Percoll density gradient separation of the non-parenchymal cells has been described in detail previously (Pulford & Souhami, 1980). For studies on morphology, enzyme content and surface membrane properties non-parenchymal cells were suspended in bicarbonate buffered RPMI 1640 (Gibco Biocult Ltd.) containing glutamine and 30% foetal calf serum (FCS, Flow) and cultured on sterile glass coverslips in 24 well Costar plates.

### *Preparation of Kupffer cells for the cytostasis experiments*

Fibronectin coated dishes were prepared from cultures of baby Hamster kidney (BHK) cells

Correspondence: K. Pulford.
Received 22 June 1984; and in revised form 9 September 1984.

© The Macmillan Press Ltd., 1985

(ICRF Laboratories) as described by Ackerman & Douglas (1978). Non-parenchymal cells from Layer II of the Percoll gradient were then added in Hepes buffered RPMI 1640 medium containing 10% FCS. After 3 h of culture at 37°C the adherent cells were removed with 3 mM ethylene diaminetetra-acetic acid (EDTA), washed and kept on ice until used (Pulford & Souhami, 1981).

*Peritoneal macrophages*

Peritoneal macrophages were used as controls for the effect of the isolation procedure as previously described (Pulford & Souhami, 1980).

*Cell morphology and cytochemistry*

May–Grunwald Giemsa (Dif-Quik, Harleco) was used for routine morphology. Non-specific esterase (NSE) was detected using the method of Yam *et al.* (1971) while peroxidase was demonstrated using a modification of the method of Kaplow (1965).

*Demonstration of Candida phagocytosis, Fc and C3 receptors*

These methods have been described in detail previously (Pulford & Souhami, 1980, 1981). *Candida guillemondeii* were used in the study of phagocytosis. Candida ($10^7$) were added to each coverslip culture. After 60 min at 37°C the percentage of cells which contained one or more Candida was counted. Sheep red cells coated with a rabbit anti-sheep IgG antibody (Flow Laboratories) were used to demonstrate the Fc receptor. Sheep cells which had only been washed were included as a control. The C3 receptors were detected using sheep red cells which had been coated with a rat anti-sheep red cell IgM antibody (received from Dr N. Hogg) prior to incubation with fresh mouse serum as a source of complement. Sheep cells incubated with only the IgM antibody were used as controls. In both of these assays 0.1 ml of a 0.5% sheep cell suspension was added to each coverslip culture. After 30 min at 37°C the number of positive cells was counted. A cell was scored positive if it contained one or more red cell or had three or more adherent red cells.

*Detection of Ia antigen*

Ascitic fluid from mice immunised with the hybridoma cell line 10-2-14 was the source of antibody directed against the I-A subregion of the Ia complex (kindly donated by Dr D. Katz). This antibody detects the I-A antigen on cells from CBA mice which are of the H-$2^k$ haplotype. Cells from BALB/c mice (H-$2^d$ haplotype) showed no positive staining with this antibody thus demonstrating its specificity for the H-$2^k$ haplotype. The method used for the detection of the Ia antigen has been described in detail before (Pulford & Souhami, 1981).

*Cytostasis assay*

The BALB/c fibroblast line K31 (KIRSTEN sarcoma virus transformed non-producer cell line, Aaronson & Weaver, 1971) was used as the target cell source in the present study. These cells were maintained as a monolayer culture in Eagles medium (Gibco Biocult Ltd.) containing 10% FCS. The method of Goldmann & Hogg (1978) was followed. After trypsinisation the target cells were suspended in bicarbonate buffered RPMI 1640 containing 10% FCS and glutamine and plated out at a concentration of $5 \times 10^3$ cells per well of a flat bottomed plastic microtitre plate (Linbro). Adherent cells from Layer II of the Percoll gradient were added to the tumour cells in concentrations to give effector:target cell ratios of 40:1 to 2:1. After overnight culture at 37°C in 5% $CO_2$ the supernatant was removed from each well and replaced with fresh medium containing 0.5 $\mu$Ci $^{125}$IUdR $ml^{-1}$ (5′-$^{125}$Iodo-2′-deoxyuridine, specific activity 5 Ci $mg^{-1}$, Radiochemical Centre, Amersham) and re-cultured for 5 h at 37°C. The plates were then washed by their total immersion 6 times in normal saline (Balkwill & Hogg, 1979). After the plates had dried they were sealed with wax and the individual wells were cut out and counted on a LKB gamma counter.

The % uptake of $^{125}$IUdR by the target cells (K31) was calculated as

$$\frac{\text{c.p.m. target} + \text{effector cells}}{\text{c.p.m. targets}} \times 100.$$

The % cytostasis effected by the Kupffer cells is therefore 100-% $^{125}$IUdR uptake.

In order to assess the cytostatic activity of culture supernatants, those supernatants which had been removed after overnight culture of the effector and target cells were added to cultures of fresh target cells and the cytostatic assay was repeated.

The effect of duration of culture of Kupffer cells on their cytostatic ability was studied by culturing adherent cells from Layer II in microtitre plates. Tumour target cells were then added after 1 and 5 days of Kupffer cell culture and the cytostasis assay was carried out as previously described.

### Results

*Isolation of Kupffer cells*

The average weight of the CBA liver was 1.14

$\pm 0.18$ g which gave a yield of $24.6 \pm 6.3 \times 10^6$ non-parenchymal cells following pronase digestion. A similar yield of $25.2 \pm 4.1 \times 10^6$ cells was obtained from the livers of BALB/c mice. Twenty-one per cent of the freshly isolated non-parenchymal cells from the CBA and the BALB/c mice had the morphological and cytochemical characteristics of Kupffer cells. After the Percoll gradient the non-parenchymal cells were separated into 3 compartments according to cell density (Pulford & Souhami, 1980, 1981). Only the cells from Layer II (specific gravity 1.055–1.080), constituting 44% of the total non-parenchymal cell population, were placed in culture. Of these 40% were mononuclear, peroxidase and NSE positive Kupffer cells.

*Characteristics of the adherent cells*

After 3 h culture 95% of the adherent cells from the cultures obtained from both BALB/c and CBA mice were judged to be peroxidase and NSE positive Kupffer cells. At this time 16% were phagocytic, 64% had Fc receptors, 14% possessed C3 receptors and 77.5% were I-A$^k$ positive. After 48 h the cells had lost their peroxidase activity but were strongly NSE positive mononuclear cells with deeply basophilic cytoplasm and the majority contained small vacuoles. Ninety-nine per cent of these cells were phagocytic and had Fc receptors while 89% possessed C3 receptors and 83.9% were I-A$^k$ positive.

This initial loss of phagocytic ability, Ia antigen and surface membrane receptors was due to the effect of pronase used during the isolation procedure. Control cultures of pronase treated peritoneal macrophages demonstrated the same changes during the first 24 h of culture but after this time gave identical results to those obtained from non-pronase treated cells.

During continued culture the Kupffer cells demonstrated a loss of their surface membrane receptors. After a total of 6 days of culture 98% of the cells were phagocytic and had Fc receptors while 83% possessed C3 receptors. All of the cells were I-A$^k$ negative. A similar loss of membrane characteristics was found in the control cultures of peritoneal macrophages.

Fifty-five per cent of the Kupffer cells placed in culture were adherent after 48 h. No further loss of adherent cells was found during a total of 6 days of culture.

*Kupffer cell cytostatic activity*

Syngeneic and allogeneic Kupffer cells exerted an equal cytostatic effect on the tumour cell lines. Addition of purified populations of Kupffer cells from BALB/c and CBA mice to the K31 cells resulted in an inhibition of $^{125}$IUdR uptake by the tumour cells. The results from a typical experiment are shown in Figure 1. At a Kupffer cell to target cell ratio of 40:1 the $^{125}$IUdR uptake by the tumour cells was decreased by more than 60%. Even at an effector:target cell ratio of 2:1 there was a 36% reduction in the $^{125}$IUdR uptake by the tumour cells. Kupffer cells possessed a greater cytostatic ability than peritoneal macrophages (Figure 1). Although Kupffer cell division has been observed after 3 days of culture (Pulford & Souhami, 1980) $^{125}$IUdR uptake by the Kupffer cells did not rise significantly above background levels. Even after a total of 6 days culture the $^{125}$IUdR uptake of the Kupffer cells was $117 \pm 10$ c.p.m. compared with $15{,}000 \pm 150$ c.p.m. in cultures of the tumour cell line.

There was no inhibition of uptake of $^{125}$IUdR by K31 cells when they were cultured in supernatants removed from the mixed cultures of tumour cells and Kupffer cells. To exclude a labile supernatant effect $5 \times 10^3$ K31 tumour cells and a mixture of Kupffer cells and tumour cells in a ratio of 40:1 were allowed to adhere to separate 6 mm sterile glass coverslips. The coverslips containing the tumour cells and the mixture of tumour cells and

**Figure 1** The cytostatic activity of cultured Kupffer cells. Each result is the mean ($\pm$s.d.) of triplicate cultures. (●——●) BALB/c Kupffer cells; (X——X) CBA Kupffer cells; (■····■) BALB/c peritoneal macrophages.

Kupffer cells were then placed in the same 16 mm well of a Costar plate. The well was flooded with media and cultured overnight. The following day the media was removed and replaced with $^{125}$IUdR. After 5 h culture the coverslips were removed, washed and counted. In the mixed culture of K31 and Kupffer cells uptake of $^{125}$IUdR was inhibited by 62% but there was no inhibition of $^{125}$IUdR uptake in the coverslip cultures of K31 cells which had been cultured without Kupffer cells in the same Costar well. This indicated the absence of a long-range labile supernatant factor and implies that cell to cell contact may be necessary for cytostasis.

*Effect of time on the cytostatic activity of cultured Kupffer cells*

A comparison of the cytostatic ability of BALB/c Kupffer cells maintained in culture for 3 h, 1 and 5 days prior to the addition of K31 tumour cells is shown in Figure 2. At an effector:target cell ratio of 40:1 the $^{125}$IUdR uptake by the tumour cells was reduced by 62% after a total culture time of 1 day, 45% after 2 days and by only 3% after a total of 6 days of Kupffer cell culture. Similar results were found for the CBA Kupffer cells.

A loss of cytostatic activity during culture was also shown by control cultures of peritoneal macrophages. This indicates that the decreased cytostatic activity found during culture was not due to any effect of the isolation procedure.

**Figure 2** The loss of cytostatic activity of BALB/c Kupffer cells during culture. Each result is the mean (±s.d.) of triplicate cultures. (●——●), (X——X) and (■——■) cytostatic activity after a total culture period of 1, 2 and 6 days respectively.

### Discussion

The recoveries and characteristics of the populations of Kupffer cells prepared here from both BALB/c and CBA mice were similar and confirmed our previous findings (Pulford & Souhami, 1980, 1981). The Kupffer cells were adherent and NSE positive. At 3 h only a minority were phagocytic and had C3 receptors, but with continued culture the majority became phagocytic and expressed both C3 and Fc receptors, all of which are characteristics of cells found capable of cytostasis in other experiments (Keller, 1973, 1976*a*; Balkwill & Hogg, 1979). The use of control cultures of peritoneal macrophages in the present study demonstrated that the pronase treatment and the Percoll gradient in the isolation procedure had no selective effect on the adherent Kupffer cell population obtained. However, since only 55% of the Kupffer cells isolated and placed in culture were adherent it remains possible that these adherent cells do represent a subpopulation of Kupffer cells.

Previous studies have shown macrophages from the spleen, lungs, breast, bone marrow and the peritoneum to be cytostatic to tumour cells (Keller, 1978; Balkwill & Hogg, 1979; Sone & Tsubura, 1982). The present study shows that liver macrophages are also capable of cytostatic activity *in vitro*. The finding that Kupffer cells exerted a greater cytostatic effect than comparable cultures of peritoneal macrophages provides evidence that cytostasis was not due solely to cell crowding. This conclusion is supported by the finding that cytostatic activity decreases as Kupffer cells are maintained in culture. Since Kupffer cells increase in size and number during culture (Pulford & Souhami, 1980) an increased inhibition of $^{125}$IUdR uptake would be expected if the results were due to cell crowding.

The cytostatic ability is not genetically restricted since Kupffer cells from CBA mice were able to inhibit $^{125}$IUdR uptake by allogeneic K31 tumour cells. The ability of allogeneic macrophages from other sites to exhibit cytostasis has previously been shown in the mouse (Hogg & Balkwill, 1981) and the rat (Keller, 1974, 1978).

The methods by which macrophages exert their cytostatic ability are still unclear. While some workers have found evidence for direct cell to cell contact (Keller, 1973; Stewart *et al.*, 1975) others have suggested that the cytostatic effect mediated by macrophages is due to the release of substances such as arginase (Currie, 1978) and tumour necrotic factors (Matthews, 1978, 1981) from the macrophages. It is also possible that in those *in vitro* experiments where isotopes such as $^{3}$H-thymidine or $^{125}$IUdR were used, that the observed cytostatic effect was due to the release by the macrophages of thymidine (Staedecker *et al.*, 1977) which inhibited the uptake of the isotope into the tumour cells. In the present study, however, the experiments with supernatants and with coverslip cultures of tumour cells and Kupffer cells provided evidence that any supernatant cytostatic effect was unlikely to be due to the presence of thymidine or any other long range non-labile factor. The possibility remains, however, that cytostasis was effected by a short range labile factor.

Evidence for heterogeneity within macrophage populations has been obtained from a variety of studies (Lee & Berry, 1977; Cowing *et al.*, 1978; Hopper *et al.*, 1979). Functional heterogeneity may exist within the Kupffer cell population. In both the present study and in a previous study (Pulford & Souhami, 1981) 89.9% of cultured Kupffer cells prepared from CBA mice were found to posses Ia antigens. It would therefore be expected that a similar proportion of Kupffer cells from BALB/c mice would be Ia positive. Since Hogg & Parish (1980) found that peritoneal exudate macrophages cytostatic for tumour cells were Ia negative it is possible that the small number of Ia negative Kupffer cells found in the present study are cytostatic. However, in the present experiments cytostatic activity is lost during culture in parallel with the Ia antigen. Cytostatic ability may therefore depend upon factors other than the presence of the Ia antigen, for example, the mode of activation of the macrophages. Tanaka *et al.* (1981) found Ia positive as well as Ia negative peritoneal exudate macrophages to be cytostatic only after their incubation with a lymphokine.

Since the decrease in cytostatic ability of cultured Kupffer cells was also found in the control cultures of peritoneal macrophages it does not seem to be an effect of the isolation procedure. Possible explanations for this loss of activity include inadequate culture conditions to maintain cytostatic activity or the loss, during culture, of surface molecules necessary for initiating cytostasis, similar to the loss of phagocytic ability, Ia antigens C3 and Fc receptors found here and in previous studies (Pulford & Souhami, 1980, 1981).

This work was supported by a grant from the Cancer Research Campaign.

**References**

AARONSON, S.A. & WEAVER, C.A. (1971). Characterization of murine sarcoma virus (KIRSTEN) transformation of mouse and human cells. *J. Gen. Virol.*, **13**, 245.

ACKERMAN, S.K. & DOUGLAS, S.D. (1978). Purification of human monocytes on microexudate-coated surfaces. *J. Immunol.*, **120**, 1372.

BALKWILL, F.R. & HOGG, N. (1979). Characteristics of human breast milk macrophages cytostatic for human cell lines. *J. Immunol.*, **123**, 1451.

BENACERRAF, B. (1964). Functions of the Kupffer cells. In: *The Liver.* (Ed. Rouiller), New York and London, Academic Press, p. 37.

COWING, C., PINCUS, S.H., SACHS, D.H. & DICKLER, H.B. (1978). A subpopulation of adherent acessory cells bearing both I-A and I-E or I-C subregion antigens is required for antigen specific murine T-lymphocytic proliferation. *J. Exp. Med.*, **121**, 1680.

CURRIE, G.A. (1978). Activated macrophages kill tumour cells by releasing arginase. *Nature*, **273**, 758.

ECCLES, S.A. & ALEXANDER, P. (1974). Macrophage content of tumours in relation to metastatic spread and host immune reaction. *Nature*, **250**, 667.

EVANS, R. (1972). Macrophages in syngeneic animal tumours. *Transplantation*, **14**, 468.

GOLDMANN, R. & HOGG, N. (1978). Enhanced susceptibility of virus-infected fibroblasts to cytostasis mediated by peritoneal exudate cells. *J. Immunol.*, **121**, 1657.

HIBBS, J.R. JR. (1974). Discrimination between neoplastic and non-neoplastic cells *in vitro* by activated macrophages. *J. Natl Cancer Inst.*, **53**, 1487.

HOGG, N. & PARISH, C.R. (1980). Surface antigens of the murine cytostatic peritoneal macrophages. *Immunology*, **41**, 187.

HOGG, N. & BALKWILL, F.R. (1981). Species restriction in cytostatic activity of human and murine monocytes and macrophages. *Immunology*, **43**, 197.

HOLTERMAN, O.A., LISAFELD, B.A., KLEIN, E. & KLOSTERGAARD, J. (1975). Cytocidal and cytostatic effects of activated peritoneal leucocytes. *Nature*, **257**, 228.

HOPPER, K.E., WOOD, P.R. & NELSON, D.S. (1979). Macrophage heterogeneity. *Vox Sang.*, **36**, 257.

KAPLOW, L.S. (1965). Simplified myeloperoxidase stain using benzidine dihydrochloride. *Blood*, **26**, 215.

KELLER, R. (1973). Cytostatic elimination of syngeneic rat tumour cells *in vitro* by non-specifically activated macrophages. *J. Exp. Med.*, **138**, 625.

KELLER, R. (1974). Modulation of cell proliferation by macrophages: a possible function apart from cytotoxic tumour rejection. *Br. J. Cancer*, **30**, 401.

KELLER, R. (1976*a*). Cytostatic and cytocidal effects in activated macrophages. In: *Immunobiology of the Macrophage*. (Ed. Nelson), New York, Academic Press, p. 487.

KELLER, R. (1976*b*). Susceptibility of normal and transformed cell lines to cytostatic and cytocidal effects by macrophages. *J. Natl Cancer Inst.*, **56,** 369.

KELLER, R. (1978). Macrophage-mediated natural cytotoxicity against various target cells *in vitro*. I. Macrophages from diverse anatomical sites and different strains of rats and mice. *Br. J. Cancer,* **37,** 732.

KRAHENBUHL, J.L. & REMINGTON, J.S. (1974). The role of activated macrophages in specific and non-specific cytostasis of tumour cells. *J. Immunol.*, **113,** 507.

LAUDER, I., AHEME, W., STEWART, J. & SAINSBURY, R. (1977). Macrophage infiltration of breast tumours: a prospective study. *J. Clin. Pathol.*, **30,** 563.

LEE, K.C. & BERRY, D. (1977). Functional heterogeneity of macrophages activated by Corynebacterium parvum: characterisation of subpopulations with different activities in promoting the immune response and suppressing tumour cell growth. *J. Immunol.*, **118,** 1530.

LOVELESS, S.E., WELLHAUSEN, S.R., BOROS, D.L. & HEPPNER, G.H. (1982). Tumouricidal macrophages isolated from liver granulomas of Schistosoma mansoni infected mice. *J. Immunol.*, **128,** 284.

LOVELESS, S.E. & HEPPNER, G.H. (1983). Tumour-associated macrophages of mouse mammary tumours. I. Differential cytotoxicity of macrophages from metastatic and non-metastatic tumours. *J. Immunol.*, **131,** 2074.

MATTHEWS, N. (1978). Tumour necrosis factor from the rabbit. II. Production by monocytes. *Br. J. Cancer,* **38,** 310.

MATTHEWS, N. (1981). Production of an anti-tumour cytotoxin by human monocytes. *Immunology,* **44,** 135.

McBRIDE, W.H., WOODRUFF, M.F.A., FORBES, G.M. & MOORE, K. (1982). Effect of C. parvum on the number and activity of macrophages in primary and transplanted murine fibrosarcoma. *Br. J. Cancer,* **46,** 448.

MOORE, K. & MOORE, M. (1977). Intra-tumour host cells of transplanted rat neoplasms of different immunogenicity. *Int. J. Cancer,* **19,** 803.

PULFORD, K. & SOUHAMI, R.L. (1980). Cell division and giant cell formation in Kupffer cell cultures. *Clin. Exp. Immunol.*, **42,** 67.

PULFORD, K. & SOUHAMI, R.L. (1981). The surface properties and antigen-presenting function of hepatic non-parenchymal cells. *Clin. Exp. Immunol.*, **46,** 581.

ROOS, F. & DINGEMANS, K.P. (1977). Phagocytosis of tumour cells by Kupffer cells *in vivo* and in the perfused mouse liver. In: *Kupffer Cells and other Liver Sinusoidal Cells.* (Eds. Wisse & Knook), Amsterdam, Elsevier/North Holland, p. 183.

RUSSELL, S.W. & McINTOSH, A.T. (1977). Macrophages isolated from regressing Moloney sarcomas are more cytotoxic than those recovered from progressing sarcomas. *Nature,* **268,** 69.

SONE, S. & FIDLER, I.J. (1980). Tumour cytotoxicity of rat alveolar macrophages activated *in vitro* by endotoxin. *J. Reticuloendo. Soc.*, **27,** 269.

SONE, S. & TSUBURA, E. (1982). Human alveolar macrophages: potentiation of their tumouricidal activity by liposome-encapsulated muramyl dipeptide. *J. Immunol.*, **129,** 1313.

STAEDECKER, M.J., CALDERON, J., KARNOVSKY, M.L. & UNANUE, E.R. (1977). Synthesis and release of thymidine by macrophages. *J. Immunol.*, **119,** 1738.

STEWART, C.C., ADLES, C. & HIBBS, J.R. JNR. (1975). Interaction of macrophages with tumour cells. *Adv. Exp. Med. Biol.*, **73B,** 423.

TALMADGE, J.E., KEY, M. & FIDLER, I.J. (1981). Macrophage content of metastatic and nonmetastatic rodent neoplasms. *J. Immunol.*, **126,** 2245.

TANAKA, H., TSURU, S. & TOKUNGA, T. (1981). Cytotoxic activity of Ia-positive macrophages enriched by fluorescence-activated cell sorting. *Immunol. Letters,* **2,** 251.

WOOD, G.W. & GILLESPIE, G.Y. (1975). Studies on the role of macrophages in regulation of growth and metastasis of murine chemically induced fibrosarcomas. *Int. J. Cancer,* **16,** 1022.

WOOD, G.W., NEFF, J.R., GOLLAHON, K.A. & GOURLEY, W.K. (1978). Macrophages in giant cell tumours of bone. *J. Pathol.*, **125,** 53.

YAM, L.T., LI, C.Y. & CROSBY, W.H. (1971). Cytochemical identification of monocytes and granulocytes. *Am. J. Clin. Pathol.*, **55,** 283.